# Inhibition of attachment of oral bacteria to immortalized human gingival fibroblasts (HGF-1) by tea extracts and tea components

**DOI:** 10.1186/1756-0500-6-143

**Published:** 2013-04-11

**Authors:** Yi Wang, Felicia FL Chung, Sui M Lee, Gary A Dykes

**Affiliations:** 1 School of Science, Monash University, Jalan Lagoon Selatan, Bandar Sunway, Selangor, 46150, Malaysia

**Keywords:** Tea, Oral bacteria, Attachment, Gingival cells, Pu-erh, Chrysanthemum

## Abstract

**Background:**

Tea has been suggested to promote oral health by inhibiting bacterial attachment to the oral cavity. Most studies have focused on prevention of bacterial attachment to hard surfaces such as enamel.

**Findings:**

This study investigated the effect of five commercial tea (green, oolong, black, pu-erh and chrysanthemum) extracts and tea components (epigallocatechin gallate and gallic acid) on the attachment of five oral pathogens (*Streptococcus mutans* ATCC 25175, *Streptococcus mutans* ATCC 35668, *Streptococcus mitis* ATCC 49456, *Streptococcus salivarius* ATCC 13419 and *Actinomyces naeslundii* ATCC 51655) to the HGF-1 gingival cell line. Extracts of two of the teas (pu-erh and chrysanthemum) significantly (p < 0.05) reduced attachment of all the *Streptococcus* strains by up to 4 log CFU/well but effects of other teas and components were small.

**Conclusions:**

Pu-erh and chrysanthemum tea may have the potential to reduce attachment of oral pathogens to gingival tissue and improve the health of oral soft tissues.

## Findings

### Introduction

Oral Streptococci such as *Streptococcus mutans* are pathogens commonly associated with dental plaque and the formation of caries [1]. In order to initiate disease these bacteria must attach to components of the oral cavity such as the enamel, tongue, saliva or gums [2].

Plant extracts and phytochemicals can inhibit bacterial attachment to abiotic and biotic surfaces by altering cell surface properties including hydrophobicity, surface charge and the presence of structures such as flagella [3-5]. Tea is one such potential attachment inhibitor [6]. Non-fermented teas or partially-fermented teas, such as green tea and oolong tea, have strong bactericidal activity and may inhibit bacterial attachment to some elements of the gastrointestinal tract [7-11]. Fully fermented teas, such as black tea and pu-erh tea, have less effective bactericidal activity but may inhibit attachment of bacteria to dental plaque [12].

Previous studies investigating bacterial attachment and inhibition by phytochemicals to components of the oral cavity have focused on attachment to hard surfaces such as enamel [10,13,14]. Attachment of bacteria to soft tissues in the mouth can also initiate disease and for this reason we investigated the effects of tea extracts and tea components on attachment of oral pathogenic bacteria to an immortalized line of connective gingival fibroblasts *in vitro*.

## Materials and methods

### Bacteria and growth conditions

Five strains of bacteria, namely *Streptococcus mutans* (ATCC 25175), *Streptococcus mutans* (ATCC 35668), *Streptococcus mitis* (ATCC 49456), *Streptococcus salivarius* (ATCC 13419) and *Actinomyces naeslundii* (ATCC 51655), were selected for this study and obtained from the American Type Culture Collection (Manassas, USA). All bacteria were maintained on Mitis Salivarius Agar (MSA; Difco, USA) at 4°C and grown in Tryptic Soy Broth (TSB; Merck, USA) at 37°C for 24 h with shaking at 150 rpm for all experiments. Bacterial suspensions were prepared by centrifuging 20 mL of TSB cultures at 7669 × g and 4°C for 15 min, washing the resultant pellet gently with phosphate buffered saline (PBS; 2.7 mM KCl, 10 mM Na_2_HPO_4_, 17 mM KH_2_PO_4_, 137 mM NaCl, pH 7.4; 1st BASE, Singapore) and resuspending it in 20 mL PBS, tea extract solutions or tea component solutions prepared as described below.

### Preparation of tea extracts and tea components

Commercial green tea, oolong tea, black tea, pu-erh tea and chrysanthemum tea (Ten Ren Tea Co. Ltd., Taiwan) extracts were prepared using 90% acetone (Sigma Aldrich, USA) at the ratio of 1:20 (wt/vol) for 2 h. The resultant extracts were evaporated under vacuum at 40°C, freeze dried and stored at −20°C until further use. Using this method reportedly allows for extraction of more than 95% of the phenolic compounds in tea, including catechins, myricetin, quercetin and kaempherol [15]. Epigallocatechin gallate (EGCg; 95% [vt/vt]; Sigma-Aldrich) and gallic acid (Sigma-Aldrich) were also used as they are major phenolic components of teas. Specifically, EGCg constitutes approximately 10% of the dry weight of green tea and its level decreases with increasing degree of fermentation [16]. Levels of gallic acid, on the other hand, increase with fermentation and constitute approximately 0.5% dry weight of black tea [17]. The stock solutions for all experiments were prepared by dissolving 100 mg of tea extracts or tea component in 10 mL PBS containing 1% (vol/vol) methanol (Systerm, UK) and the resultant solutions were filter sterilized though a 0.2 μm filter (Millipore, USA).

### Determination of total phenolic, total tannin and total flavonoid content

Total phenolic and total tannin contents of the tea extracts were determined using the Folin-Ciocalteau colorimetric method [18]. To determine the total phenolic content, a 15 μL tea extract solution (1 mg/mL) was added to 80 μL of 7.5% (wt/vol) sodium carbonate (R&M Chemicals, Malaysia) and 75 μL of 10% (vol/vol) Folin-Ciocalteau reagent (R&M Chemicals) in a well of a microtitre plate (Jet Biofil, China). The plate was incubated in the dark for 30 min before measuring the absorbance at 765 nm. To determine the total tannin content, 0.5 mL of the sample solution was mixed with 0.5 mL of distilled water and 50 mg of poly(vinylpolypyrrolidone) (PVPP; Sigma-Aldrich) which has a high affinity to tannins. The mixture was vortexed, incubated at 4°C for 15 min and vortexed again prior to centrifuging at 1409 g for 10 min in order to remove tannins. The supernatant containing non-tannin phenolics was then quantified using the Folin-Ciocalteau method described above. The difference between the total phenolic content and the non-tannin phenolic content is the measure of tannins. A standard curve was plotted using gallic acid, and the total phenolic and total tannin contents were expressed as μg gallic acid equivalent (GAE) / mg.

Total flavonoid content was measured using the aluminum chloride colorimetric method [19]. A 50 μL of tea extract sample dissolved in methanol (1 mg/mL) was added to 10 μL of 10% (wt/vol) aluminum chloride (Bendosen, Malaysia), 10 μL of 1 M potassium acetate (R&M Chemicals) and 80 μL of distilled water in the wells of a microtitre plate. The plate was incubated at room temperature for 30 min before the absorbance was measured at 435 nm. The blank was prepared using distilled water in place of aluminum chloride. A standard curve was plotted using quercetin (Sigma-Aldrich) and the total flavonoid content was expressed as μg quercetin equivalent (QE) / mg.

### Cell culture

Immortalized human gingival fibroblast-1 HGF-1 (ATCC CRL-2014) were obtained from the American Type Culture Collection and cultured in high glucose Dulbecco’s modified Eagle’s medium (DMEM; Sigma-Aldrich) supplemented with 4 mM L-glutamine (Sigma-Aldrich) and 10% (vol/vol) heat-inactivated fetal bovine serum (FBS; Sigma-Aldrich). No antibiotic supplement was used. Cells were incubated at 37°C in 5% CO_2_ atmosphere, fed every 48 h and routinely sub-cultured every 5 days with a split ratio of 1:3 using 1 × trypsin-EDTA (0.05%; Sigma-Aldrich) for 3 min at 37°C.

### Bacterial attachment assay

Bacterial attachment assays were as described by Mellor, Goulter, Chia, and Dykes [20] with some modifications. Briefly, monolayers of HGF-1 cells were grown in 24-well tissue culture plates (Jet Biofil) to a density of 1.8 (±0.2) × 10^5^ cells per well (approximately 100% coverage). Prior to the attachment assay the culture medium in each well was removed and the cell monolayer was washed with PBS. The monolayer was incubated at 37°C for 30 min with 2 mL aliquots of tea extracts or tea components (PBS as control) containing suspended bacteria (~1 × 10^7^ CFU/mL). The concentrations of the tea extracts and compounds used to suspend bacteria were previously determined by antimicrobial susceptibility assays and cytotoxicity assays not kill or inhibit the bacteria or the HGF-1 cells at the concentrations used in this study. After incubation the supernatant in each well was removed and the wells were washed three times with 2 mL PBS. The monolayer with bacteria attached was then detached by incubating with 400 μL 0.3 × trypsin-EDTA (at which concentration trypsin does not kill or inhibit the bacteria) at 37°C for 5 min. The detached bacteria were then serial diluted, spread plated on Tryptic Soy Agar (TSA; Merck) and quantified after 24 h incubation. The ability of bacteria to attach to wells without HGF-1 cells was also determined in order to ensure that the bacteria attached to HGF-1 cells but not to the plastic material of the plate. The numbers of bacteria attached to the cell line was expressed as log CFU/well.

### Statistical analysis

A one way ANOVA (Tukey’s comparison) was performed on all data sets using MINITAB software (MINITAB 15.1; Minitab Inc., USA) at a 95% confidence level. All assays were performed in triplicate with independently grown cultures.

## Results and discussion

The results of total phenolic, total tannin and total flavonoid content assays are presented in Table 1. The total phenolic and total flavonoid content decreased and total tannins increased, with an increasing degree of fermentation from green tea to oolong tea to black tea to pu-erh tea. These differences between teas are probably due to the polymerization of flavonoids (especially flavon-3-ols) into large molecule polyphenols (tannins) which occur during the fermentation process [16]. Chrysanthemum tea, which is a blend of black tea and dried chrysanthemum, had similar levels of total phenolic and total tannin to pu-erh tea and similar levels of flavonoids to green tea. This suggests that dried chrysanthemum is rich in flavonoids.

**Table 1:** Total phenolic, total tannin and total flavonoid contents of the tea extracts

	**Total phenolic content (μg GAE / mg)**	**Total tannin content (μg GAE / mg)**	**Total flavonoid content (μg QE / mg)**
Green tea	527 ± 34 (a)	149 ± 26 (a)	7.30 ± 0.68 (a)
Oolong tea	469 ± 28 (a, b)	161 ± 35 (a, b)	4.89 ± 0.14 (b)
Black tea	411 ± 20 (b, c)	241 ± 19 (b, c)	2.97 ± 0.59 (c)
Pu-erh tea	349 ± 35 (c)	305 ± 34 (c)	1.68 ± 0.68 (c)
Chrysanthemum tea	376 ± 13 (c)	280 ± 8 (c)	7.61 ± 0.42 (a)

All results are presented as the means followed by SDs. Values labeled with the same letter are not significantly different (p > 0.05) among the tea extract samples. Tukey’s comparisons were conducted separately for each assay.

Baseline data for attachment of the bacterial strains to the cell line and empty wells are shown in Table 2. Bacterial attachment to the cell line was ~2 log higher (p < 0.05) than that to the plastic in the wells indicating that 90% to 99% of bacteria were attached to the cell line and validating the assay.

**Table 2:** Baseline data for bacterial attachment to the HGF-1 cell line and empty wells

	**Mean±SD attachment (log CFU/well)**				
	***Streptococcus mutans *ATCC25175**	***Streptococcus mutans *ATCC35668**	***Streptococcus salivarius *ATCC13419**	***Streptococcus mitis *ATCC49456**	***Actinomyces naeslundii *ATCC51655**
Attachment to cell line	4.67 ± 0.25	4.75 ± 0.35	3.68 ± 0.77	5.11 ± 0.14	4.68 ± 0.34
Attachment to empty wells	2.37 ± 0.18	2.38 ± 0.27	2.14 ± 0.49	2.34 ± 0.47	2.19 ± 0.08

**Figure 1:**
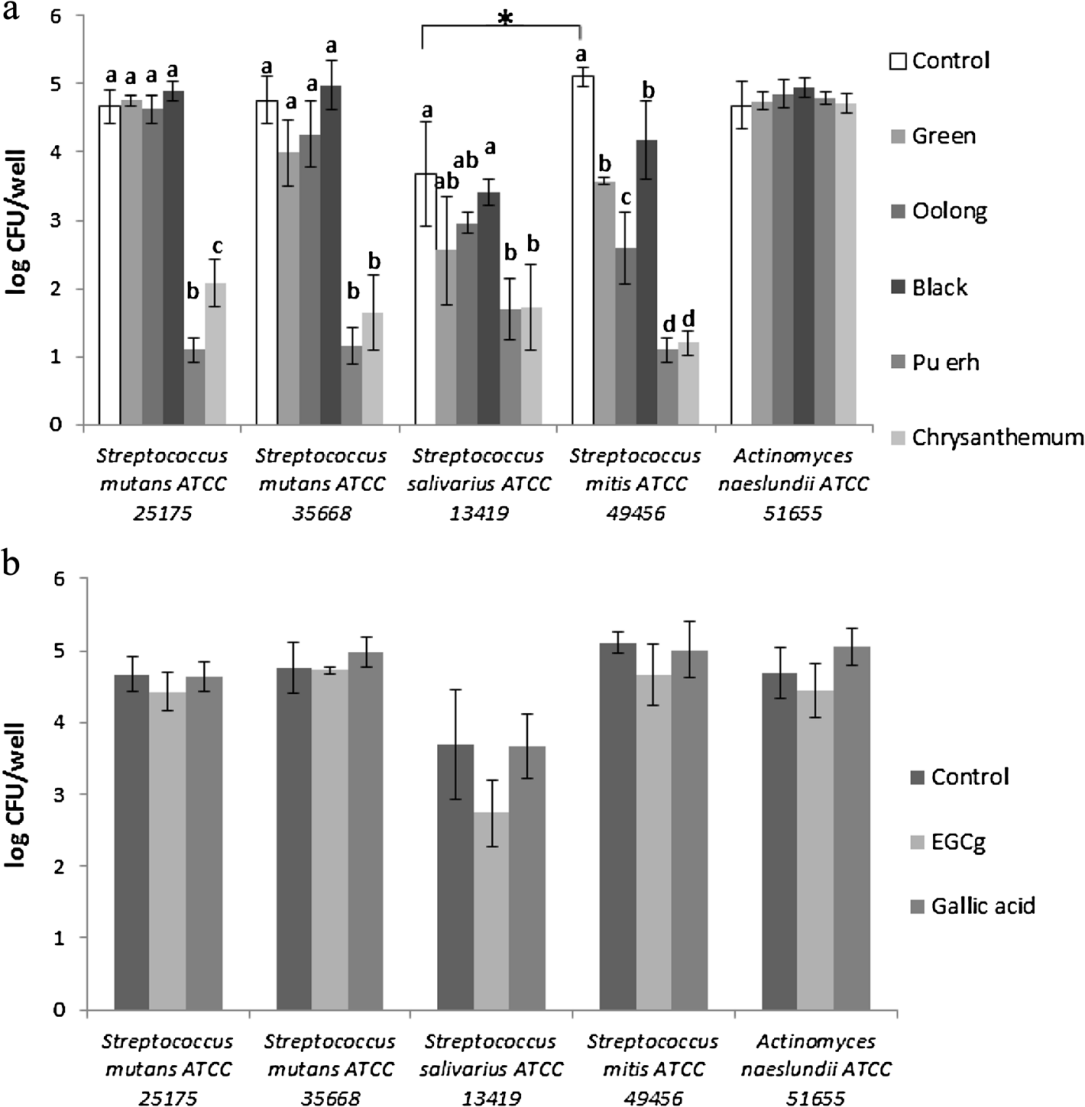
**Effects of extracts and compounds on oral bacterial attachment.** Effect of tea extracts (**a**) and EGCg and gallic acid (**b**) on oral bacterial attachment to the HGF-1 gingival cell line (log CFU/cm^**2**^, n = 3). Values labeled with the same letter are not significantly different (p > 0.05) among the treatments within a strain. Tukey’s comparisons were conducted separately for each strain. The * symbol indicates that the attachment of *Streptococcus salivarius* was significantly different from that of *Streptococcus mitis* (p < 0.05).

The results of assays investigating the effect of the tea extracts and tea components on bacterial attachment to the cell line are presented in Figure 1. All strains exhibited a similar ability to attach to the cell line except for *Streptococcus salivarius* ATCC 13419 which attached in significantly lower numbers as compared to *Streptococcus mitis* ATCC 49456 (p < 0.05). Green tea extracts, oolong tea extracts and black tea extracts inhibited the attachment of *Streptococcus mitis* ATCC49456 by between ~1 and ~2 log CFU/well (90-99% attachment inhibition; p < 0.05), but had no effect on the other strains (p > 0.05). Pu-erh tea extracts and chrysanthemum tea extracts, on the other hand, reduced the attachment of all *Streptococcus* strains to cells by between ~2 and ~4 log CFU/well (99–99.99% attachment inhibition; p < 0.05). The attachment of *Actinomyces naeslundii* ATCC 51665 to cells was not affected by any of the tea extracts tested (p > 0.05). Of particular note is that the extract of chrysanthemum tea, which, as mentioned above, is a blend of black tea and dried chrysanthemum, had a greater (p < 0.05) effect on inhibiting attachment than the black tea extract alone. This suggests that the active compounds in the chrysanthemum tea extract were contributed by the chrysanthemum components and not the black tea components of the mix. Pu-erh tea and chrysanthemum tea extracts, which were found to contain relatively higher levels of tannin, had a greater effect (p < 0.05) than the non-fermented or partially-fermented tea extracts on *Streptococcus* strains, suggesting that Streptococci may be more sensitive to polymeric flavonoids or other large molecule polyphenols with respect to their attachment to HGF-1.

Non-fermented or partially-fermented teas, such as green tea and oolong tea, have been previously shown to inhibit the attachment of *Streptococcus mutans* to collagen and tooth surfaces [12]. As indicated, in our study extracts of these teas only slightly inhibited the attachment of one bacterial strain to the gingival cell line. In addition, EGCg and gallic acid were found to have no significant effect (p > 0.05) on the ability of all strains to attach to the cell line. This finding suggests a possible reason for the relative ineffectiveness of the lower degree fermented tea extracts (green tea and oolong tea), which are rich in these compounds, in inhibiting adhesion. Fibronectin (Fn) is located on the outer surface of the HGF-1 plasma membrane and acts as a receptor protein for oral bacteria such as *Treponema denticola*[21]. *Streptococcus mutans* and *Streptococcus salivarius* have wall-associated protein A (wapA) in their outer membrane that allows them to bind collagen and a wide range of extracellular matrix molecules including type I collagen, laminin, keratin and Fn [22,23]. Tea catechins, such as EGCg, have been reported to impair the adhesion promoting ability of Fn [24], and inhibit the interactions between Fn and attaching cells by binding to the Fn receptor integrin β1 [25]. These catechins should theoretically inhibit attachment but this was not the case in our study.

This study suggests that the mechanisms of inhibition of attachment of oral pathogens to gingival cells by tea or tea extracts may be different than that of inhibition to other components of the oral cavity. Based on this *in vitro* study extracts of pu-erh tea and chrysanthemum tea, in particular, may have the potential to reduce attachment of oral pathogens to gingival tissue and improve the health of oral soft tissues but this finding needs to be confirmed by *in vivo* studies. In order to further assess the situation in the oral cavity testing fresh brewed teas (hot water extracts) for adhesion inhibitory effect is required. The experimental setup used in this study could also be used to evaluate the effect of tea on the adhesion of other oral pathogenic microorganism, such as *Candida albicans*, which have been reported to adhere to human buccal epithelial cells and cause oral candidosis [26].

## Abbreviations

HGF-1: Human gingival fibroblast-1; CFU: Colony forming unit; ATCC: American type culture collection; PBS: Phosphate buffered saline; EGCg: Epigallocatechin gallate; TSA: Tryptic soy agar; TSB: Tryptic soy broth; PVPP: Poly(vinylpolypyrrolidone); GAE: Gallic acid equivalent; QE: Quercetin equivalent; DMEM: Dulbecco’s modified Eagle’s medium; FBS: Fetal bovine serum; ANOVA: Analysis of variance; Fn: Fibronectin; WapA: Wall-associated protein A; SD: Standard deviation.

## Competing interests

The authors declare that they have no competing interests.

## Authors’ contributions

YW carried out the cell culture, attachment study, statistical analysis and drafted the manuscript. FFLC participated in the cell culture and reviewed the manuscript. SML reviewed and revised the manuscript for intellectual content. GAD contributed to the conception and design of the study and reviewed and revised the manuscript. All authors read and approved the final manuscript.
